# Melatonin Has the Potential to Alleviate Cinnamic Acid Stress in Cucumber Seedlings

**DOI:** 10.3389/fpls.2017.01193

**Published:** 2017-07-13

**Authors:** Juanqi Li, Yang Li, Yongqiang Tian, Mei Qu, Wenna Zhang, Lihong Gao

**Affiliations:** Beijing Key Laboratory of Growth and Developmental Regulation for Protected Vegetable Crops, China Agricultural University Beijing, China

**Keywords:** melatonin, cinnamic acid, cucumber, morphology, nutrient element, endogenous hormone

## Abstract

Cinnamic acid (CA), which is a well-known major autotoxin secreted by the roots in cucumber continuous cropping, has been proven to exhibit inhibitory regulation of plant morphogenesis and development. Melatonin (MT) has been recently demonstrated to play important roles in alleviating plant abiotic stresses. To investigate whether MT supplementation could improve cucumber seedling growth under CA stress, we treated cucumber seeds and seedlings with/without MT under CA- or non-stress conditions, and then tested their effects on cucumber seedling growth, morphology, nutrient element content, and plant hormone. Overall, 10 μM MT best rescued cucumber seedling growth under 0.4 mM CA stress. MT was found to alleviate CA-stressed seedling growth by increasing the growth rates of cotyledons and leaves and by stimulating lateral root growth. Additionally, MT increased the allocation of newly gained dry weight in roots and improved the tolerance of cucumber seedlings to CA stress by altering the nutrient elements and hormone contents of the whole plant. These results strongly suggest that the application of MT can effectively improve cucumber seedling tolerance to CA stress through the perception and integration of morphology, nutrient element content and plant hormone signaling crosstalk.

## Introduction

Cucumber (*Cucumis sativus* L.) is a worldwide cultivated crop and China accounts for about 77% of the global production (FAO, 2016). The intensive cultivation applied in the production systems in China leads to affect negatively the plant growth through a phenomenon of autotoxicity (Bennett et al., 2012).

In general, autotoxicity results from the presence of various autotoxins secreted by roots (Yu and Matsui, 1994; Zeng, 2014). Among them, Cinnamic acid (CA) is one of the major compound which can inhibit cucumber growth (Yu and Matsui, 1994). Qiao (2013) found that CA at 0.25 mM determined a growth inhibition on cucumber seeds by reducing the germination efficiency and affecting the further development of seedlings (reduction of growth rate of roots and less lateral roots). This was found to be correlated to several CA-induced negative impacts such as, decreased ion uptake and transport, disruption antioxidant system, and imbalanced hormonal regulation. In addition, it has been proved that CA can affect the chlorophyll content in leaves and decreases photosynthetic rate (Baziramakenga et al., 1995).

Beside the importance on animal life (Nawaz et al., 2016), there is a growing evidence that melatonin (MT, N-acetyl-5-methoxytryptamine) plays a role in alleviating plant abiotic stresses, such as, extreme temperature (Fan et al., 2015), excess copper (Zhang et al., 2014), salinity (Li et al., 2017), drought (Zhang et al., 2013), and alkaline stress (Liu N. et al., 2015). The beneficial effects of MT have been proved for the improvement of several stages of the plant life cycle, such as, germination (Aguilera et al., 2015), cell division (Park and Back, 2012), root development (Passaia et al., 2014; Wang Q. N. et al., 2016), leaf senescence (Lee and Back, 2017), and crop production (Wei et al., 2014). In addition, it has been proved that MT efficiently regulates antioxidant defense systems (Szafrańska et al., 2016), increases photosynthetic efficiency (Zheng et al., 2017), reduces chlorophyll degradation, and delays leaf senescence (Liang et al., 2015) under abiotic stresses.

Cucumber (*C. sativus* L.) is an economically important crop in China, but it is generally sensitive to CA stress (Qiao, 2013). Although numerous recent studies have demonstrated that MT plays important roles in alleviating abiotic stresses in cucumber seedlings, such as, NaCl stress (Zhang et al., 2014; Wang L. Y. et al., 2016), water stress (Zhang et al., 2013), chilling stress (Balabusta et al., 2016; Zhao et al., 2016), high temperature stress (Xu, 2010), oxidative stress (Li et al., 2016), and nitrate stress (Zhang et al., 2017), little information is available regarding the effects of MT on plant growth under CA stress. In this study, therefore, we treated cucumber seeds and seedlings with/without MT under CA- or non-stress conditions, and then assessed cucumber seedling growth, morphology, and nutrient element and plant hormone content. The aims of this study were (1) to determine if MT pretreatment can alleviate the effects of CA stress on cucumber seedlings and (2) to detect the main factor induced by MT that alleviates inhibition of cucumber seedling growth under CA stress.

## Materials and methods

### Plant materials and growth conditions

Cucumber (*C. sativus* L. cv. Jinyou No. 35) seedlings were chosen as plant material for the further experiments. Seeds were surface sterilized by a 3-min dip in NaOCl solution (3% available chloride) followed by ethanol 70% (v/v) for 1.5 min. Subsequently the seeds were washed four times with sterilized distilled water. After the sterilization procedure, cucumber seeds were treated with a MT solution (200 mL) at different concentrations (experiment 1: 0.5, 1 and 10 μM; experiment 2: 10 μM) for 6 h and incubated at 28°C. Seeds treated at the same temperature with only water served as control. Then, seeds were transferred to a Petri dish (25 seeds/Petri dish) filled with 6 mL of MT solution at the same concentration applied during the above mentioned treatments after sterilization. Seeds untreated with MT served as control. Seedlings (with a fully developed true leaf and a newly unfolded young leaf) were transferred onto hydroponic devices (Wang X. Z. et al., 2016; three seedlings/device) filled with 5 L of a nutrient solution (Yamazaki, 1982; full-strength) supplemented with MT at the same concentrations applied at seed stage. Seedlings growing on nutrient solution with no MT provided the control treatment. The culture conditions were: 28°C, under darkness for 30 h, then under light (250 μmol photons m^−2^ s^−1^, 12-h photoperiod) at 26/18°C (day/night) for 17 days. After this cultural period, the following experiments 1 and 2 were carried out.

### The effect of MT on CA treatments

#### Experiment 1

To determine the appropriate concentration of CA that could obviously inhibit seedling growth and the appropriate concentration of MT that could efficiently alleviate CA-stress in seedlings, we first conducted a two-factor experiment consisted of four MT concentrations (0, 0.5, 1, and 10 μM) and four CA concentrations (0, 0.1, 0.2, and 0.4 mM). A total of 16 treatments were conducted (4 MT concentrations × 4 CA concentrations). Each treatment had three replicates and each of the three replicates had six seedlings. The seedlings were pre-treated with/without MT using the methods described above, and subsequently subjected to CA treatments by adding CA to nutrient solution. Seedlings growing on nutrient solution with no CA provided the control treatment. The culture conditions for seedlings under CA treatments were: 26°C/18°C (day/night), under light (250 μmol photons m^−2^ s^−1^, 12-h photoperiod) for 5 days. On days 0 and 5 after CA treatments, the seedlings (three seedlings from each replicate at each sampling time) were sampled and separated into stem, leaf, and root with a sterilized scalpel. Leaf area and the dry weights of stem, leaf, and root were measured using the methods described below.

#### Experiment 2

Based on the results of experiment 1, seedling growth was obviously inhibited by 0.4 mM CA under non-MT conditions (compare 0 μM MT/0.4 mM CA vs. 0 μM MT/0 mM CA) and this growth inhibition was efficiently alleviated by 10 μM MT (compare 10 μM MT/0.4 mM CA vs. 0 μM MT/0.4 mM CA). Therefore, to comprehensively investigate the role of MT in alleviating CA stress in seedlings, we further conducted a two-factor experiment consisted of two MT concentrations and two CA concentrations. The two MT concentrations were 0 μM (−MT) and 10 μM (+MT). The two CA concentrations were 0 mM (−CA) and 0.4 mM (+CA). The combinations of the treatments were −MT/−CA (control), −MT/+CA, +MT/−CA, and +MT/+CA. Each treatment was replicated three times and each of the three replicates had nine seedlings. The seedlings were pre-treated with MT followed by CA treatments using the same methods described for experiment 1. On days 0 and 5 after CA treatments, the seedlings (three seedlings from each replicate on day 0 and 6 seedlings on day 5, respectively) were sampled and separated into root, stem, cotyledon, 1st true leaf, 2nd true leaf, and 3rd true leaf with a sterilized scalpel. Leaf area, root morphology, and the dry weights of stem, leaf, and root were measured using the methods described below. In addition, the contents of nutrient elements and endogenous phytohormones in all seedling tissues were also measured.

### Measurement of dry weight, leaf area, and root morphology

In both experiments 1 and 2, stems, leaves, and roots were dried at 75°C for 2 d and weighed to estimate the dry weight. Fresh leaves and roots were scanned (Expression 4990, Epson, Long Beach, CA), and the leaf area, root length, root diameter, root surface area, and root volume were quantified with computer image-analysis software (Win RHIZO, Régent Instruments Inc., Canada). Additionally, in experiment 2, the main root path length, apical zone length, lateral root system size, lateral root density/main root, and lateral root-related parameters (Table 1, Supplementary Figure S1) were calculated with ImageJ software (V1.50b) (Abràmoff et al., 2004) as described by Kellermeier et al. (2014).

**Table 1:** Root morphological characteristics quantified in this study.

**Abbreviation**	**Unit**	**Description**
MR		Main root
LR		Lateral root
TRS	cm	Sum of path length of the MR and LRs
MRP	cm	MR path length
Apical	cm	MR path length from last LR to MR tip
1st LRS	cm	Sum of path length of the first-order LRs
1st order LR no.		Number of first-order LRs (emerging from the MR)
2nd LRS	cm	Sum of path length of the second-order LRs
2nd order LR no.		Number of second-order LRs (emerging from first-order LRs)
1/4 LRS	cm	Sum of path length of the first-order LRs in basal quarter of MR(0–25% of the MR)
1/4 1st order LR no.		Number of first-order LRs in basal quarter of MR (0–25% of the MR)
1/4 LRP	cm	Mean LR path length in basal quarter of MR(0–25% of the MR)
1/2 LRS	cm	Sum of path length of the first-order LRs in second quarter of MR(25–50% of the MR)
1/2 1st order LR no.		Number of first-order LRs in second quarter of MR (25–50% of the MR)
1/2 LRP	cm	Mean LR path length in basal quarter of MR(25%-50% of the MR)
LRS	cm	Sum of path length of LRs
LR density/MR	cm^−1^	1st order LR no. divided by MRP

### Calculation of the growth rates of dry weight and leaf area

In both experiments 1 and 2, the relative growth rate (RGR), unit leaf ratio (ULR), average growth rate (AGR), and specific leaf area (SLA) were calculated as follows (Hunt, 1978):

(1) $$\begin{array}{c} \text{RGR}=\frac{\text{ln}\;W_{2}-\text{ln}\;W_{1}}{T_{2}-T_{1}} \end{array}$$

(2) $$\begin{array}{c} \text{ULR}=\frac{W_{2}-W_{1}}{T_{2}-T_{1}}\cdot \frac{\text{ln}\;L_{A2}-\text{ln}\;L_{A1}}{L_{A2}-L_{A1}} \end{array}$$

(3) $$\begin{array}{c} \text{AGR}=\frac{W_{2}-W_{1}}{T_{2}-T_{1}} \end{array}$$

(4) $$\begin{array}{c} \text{SLA}=\frac{L_{A}}{W_{L}} \end{array}$$

where *W*_1_ and *L*_*A*1_ present the dry weight and leaf area at time *T*_1_ (day 0 after CA treatments), respectively, *W*_2_ and *L*_*A*2_ are the dry weight and leaf area at time *T*_2_ (day 5 after CA treatments), respectively, and *W*_*L*_ is the leaf dry weight.

### Analysis of nutrient element contents in plants

In experiment 2, to measure the contents of nutrient elements, seedling tissues (root, stem, cotyledon, 1st true leaf, 2nd true leaf, and 3rd true leaf) were dried at 75°C for 2 d and then ground into to fine powder using a mortar and pestle. Approximately 0.25 g dried and ground sample was used to measure the contents of nutrient elements. The contents of C and N in seedling tissues were measured by combustion at 900°C followed by analysis using an elemental analyzer (vario PYRO cube, Germany). The contents of P, K, Ca, Mg, Fe, Mn, Zn, and Cu in seedling tissues were also determined after microwave-assisted nitric acid digestion followed by analysis using inductively coupled plasma atomic emission spectrometry (ICP-AES) (Jones et al., 1991).

### Measurement of endogenous phytohormone contents

In experiment 2, to measure the contents of endogenous phytohormones, approximately 0.5 g fresh seedling tissues (root, stem, cotyledon, 1st true leaf, 2nd true leaf, and 3rd true leaf) was frozen in liquid nitrogen and ground into to fine powder using a mortar and pestle, followed by extraction with 4 mL 80% (v/v) methanol containing 1% (v/v) butylated hydroxytoluene at 4°C for 12 h. Phytohormones, including indole-3-acetic acid (IAA), abscisic acid (ABA), gibberellic acid (GA_3_), methyl jasmonate (MeJA), and zeatin riboside (ZR) were measured using enzyme-linked immunosorbent assay (ELISA) as described in Chen et al. (2009).

### Statistical analysis

Statistical analysis was carried out with SPSS 22.0 (SPSS Inc., Chicago, USA). In both experiments 1 and 2, multiple comparisons using Tukey's honestly significant difference (HSD) *post-hoc* test were done whenever the analysis of variance (ANOVA) indicated significant differences (*P* ≤ 0.05). Additionally, in experiment 2, all data were also analyzed by two-way ANOVA with the factors being cinnamic acid (CA), melatonin (MT), and the interaction of CA × MT.

In experiment 2, the radar chart was used to visualize the effects of CA treatments on root parameters under MT or non-MT conditions. For treatments −CA/−MT and +CA/–MT, the mean of each root parameter on day 5 after CA treatments was normalized to the mean of the same parameter measured under non-MT (−MT) condition on day 0 after CA treatments. For treatments −CA/+MT and +CA/+MT, the mean of each root parameter on day 5 after CA treatments was normalized to the mean of the same parameter measured under MT (+MT) condition on day 0 after CA treatments.

In experiment 2, principal component analysis (PCA) was performed with R software (3.3.1) to comprehensively determine the relationships between morphological characteristics, nutrient element contents, endogenous hormone contents, and treatments. Pearson correlations were run among variables and Pearson's correlation coefficients (*r*) were derived using SPSS 22.0.

## Results

### The appropriate MT concentration efficient in alleviating CA stress

**Figure 1:**
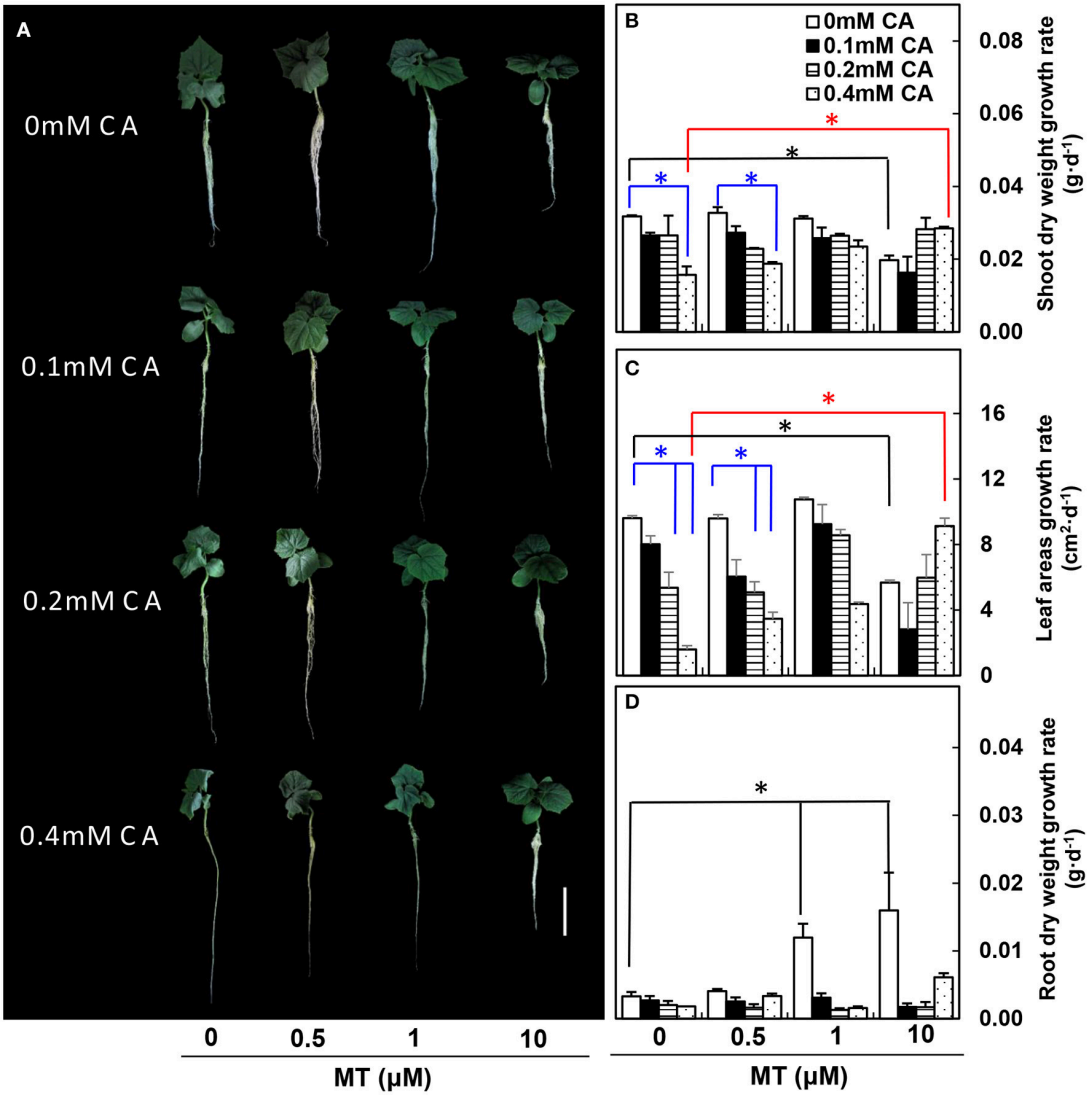
The MT-pretreated cucumber seedlings at different CA concentrations. **(A)** Morphological changes of cucumber seedlings. **(B)** Shoot dry weight growth rates. **(C)** True leaf areas growth rates. **(D)** Root dry weight growth rates. Values are means ± standard error (*n* = 3). Color-coded lines and asterisks indicate significant differences. ^*^Indicates significance at *P* < 0.05 by Tukey's HSD-test. Bar = 10 cm.

In experiment 1, under non-MT (0 μM) conditions, the cucumber seedling growth was suppressed gradually as CA concentration increased, and cucumber seedlings showed obvious wilting symptoms at 0.4 mM CA (Figure 1A). In particular, under non-MT (0 μM) conditions, the growth rates of both shoot dry weight and leaf area were significantly lower at 0.4 mM CA than at 0 mM CA (blue asterisks; Figures 1B,C). However, the negative effects of 0.4 mM CA were efficiently reduced by 10 μM MT (compare 10 μM MT/0.4 mM CA vs. 0 μM MT/0.4 mM CA, red asterisks; Figures 1B,C). The growth rate of root dry weight was not influenced by CA under non-MT (0 μM) conditions, but was significantly higher at 1 and 10 μM MT than at 0 μM MT under non-CA (0 mM) conditions (black asterisks; Figure 1D).

### MT improves the growth of leaves and roots under CA stress

**Figure 2:**
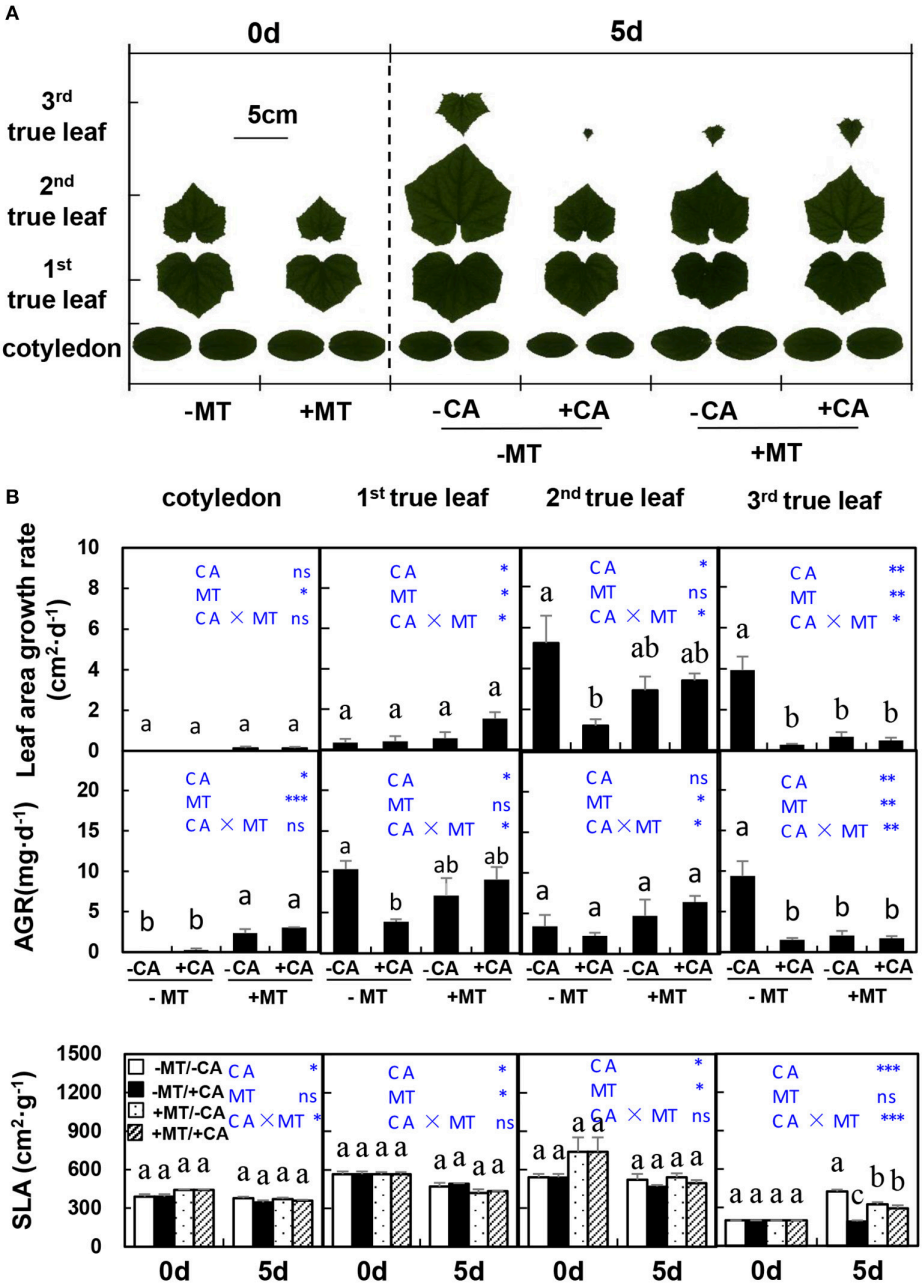
The leaf morphological characteristics of cucumber seedlings as affected by MT and CA treatments. **(A)** Scanned images of leaves. **(B)** Leaf area growth rates, dry mass average growth rate (AGR) and specific leaf area (SLA) of cotyledon and the 1st, 2nd, and 3rd true leaves. –MT and +MT represent application of 0 and 10 μM MT, respectively. −CA and +CA indicate application of 0 and 0.4 mM CA, respectively. Values are means ± standard error (*n* = 3). Different letters over the bars denote significance at *P* < 0.05 by Tukey's HSD-test. Light-blue letters and asterisks indicate the sources of variation: CA, cinnamic acid; MT, melatonin; CA × MT, the interaction of CA and MT. ^*^P < 0.05; ^**^P < 0.01; ^***^P < 0.001; ns: not significant.

In experiment 2, on day 5 after CA treatments, the areas of all true leaves and cotyledon were obviously decreased by CA under non-MT conditions (compare −MT/+CA vs. −MT/−CA), but were not influenced by CA under MT conditions (compare +MT/+CA vs. +MT/−CA), indicating the role of MT in alleviating CA stress (Figure 2A). Similar trends were observed in the SLA of 3rd true leaf (Figure 2B). Moreover, the leaf area growth rate and AGR of the 2nd true leaf were significantly reduced by −MT/+CA but were not affected by +CA/+MT, when compared to −MT/−CA, further demonstrating the alleviating role of MT in CA stress.

**Figure 3:**
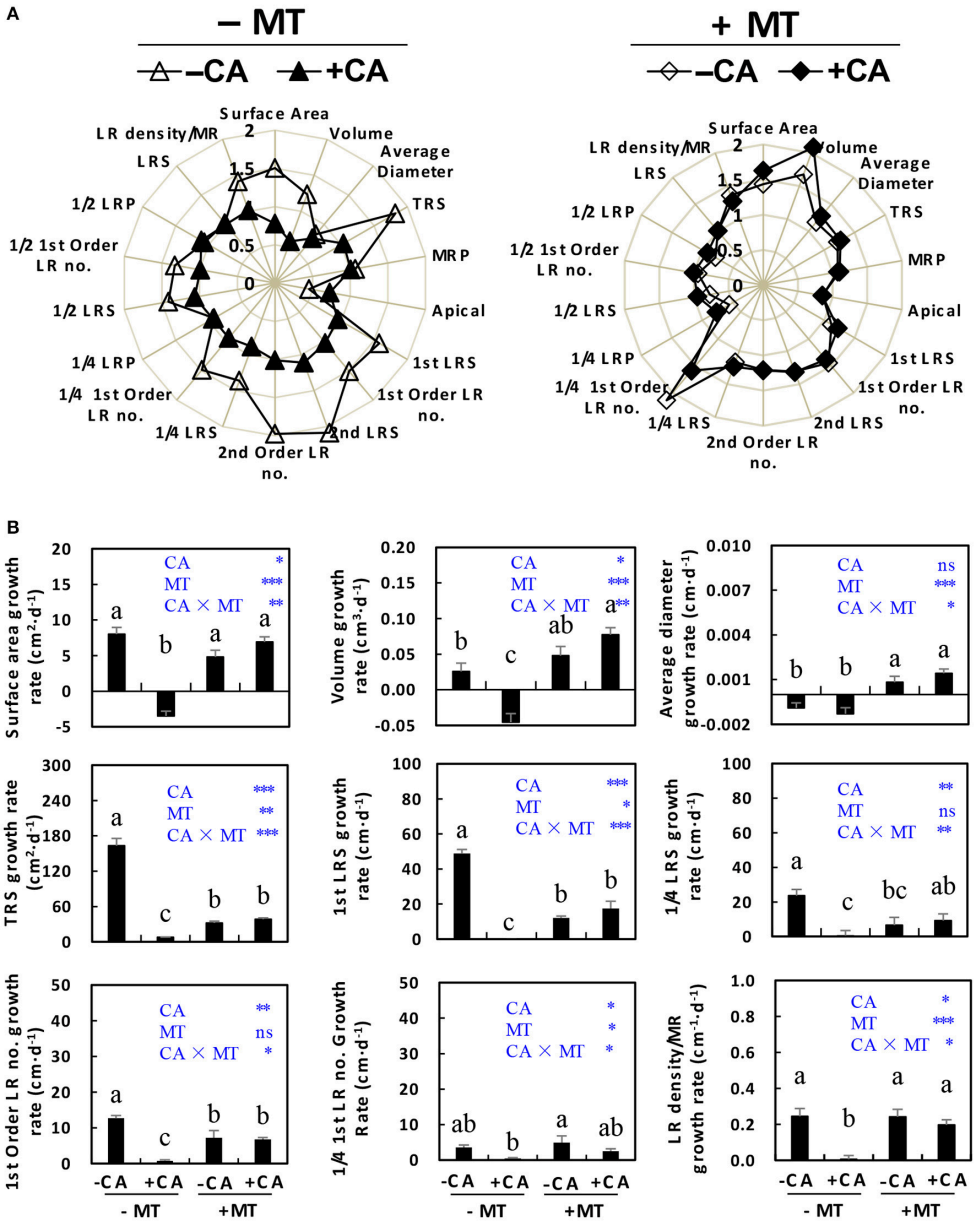
The root morphological characteristics of cucumber seedlings as affected by MT and CA treatments. **(A)** Effects of CA on overall root morphology under conditions with/without MT, visualized as radar charts. **(B)** The growth rates of surface area, root volume, average diameter, TRS, 1st LRS, 1/4 LRS, 1st Order LR no., 1/4 1st Order LR no., and LR density/MR. For definitions of these root parameters, see Table 1 and Supplementary Figure S1. −MT and +MT represent application of 0 and 10 μM MT, respectively. −CA and +CA indicate application of 0 and 0.4 mM CA, respectively. Values are means ± standard error (*n* = 3). Different letters over the bars denote significance at *P* < 0.05 by Tukey's HSD-test. Light-blue letters and asterisks indicate the sources of variation: CA, cinnamic acid; MT, melatonin; CA × MT, the interaction of CA and MT. ^*^P < 0.05; ^**^P < 0.01; ^***^P < 0.001; ns: not significant.

The root growth was also generally improved by MT under CA stress. Table 1 shows the abbreviations for root growth parameters which are used further on to assess this statement. The radar chart (Figure 3A) showed that most root parameters (e.g., surface area, volume, TRS, 1st LRS, 1st order LR no., 2nd LRS, 2nd order LR no, 1/4 LRS, 1/4 1st order LR no., 1/2 LRS, 1/2 1st order LR no., and LR density/MR) were obviously reduced by CA under non-MT conditions (compare −MT/+CA vs. −MT/−CA), but were slightly influenced by CA under MT conditions (compare +MT/+CA vs. +MT/−CA). Specifically, the −MT/+CA treatment significantly decreased the growth rates of root surface area, volume, TRS, 1st LRS, 1/4 LRS, 1st order LR no., and LR density/MR compared to −MT/−CA (Figure 3B). However, the negative effects of CA on most root parameters were significantly reduced by MT (compare +MT/+CA vs. −MT/+CA), suggesting that MT enhanced root growth under CA stress (Figure 3B).

### MT increases the dry matter accumulation of seedlings under CA stress

**Figure 4:**
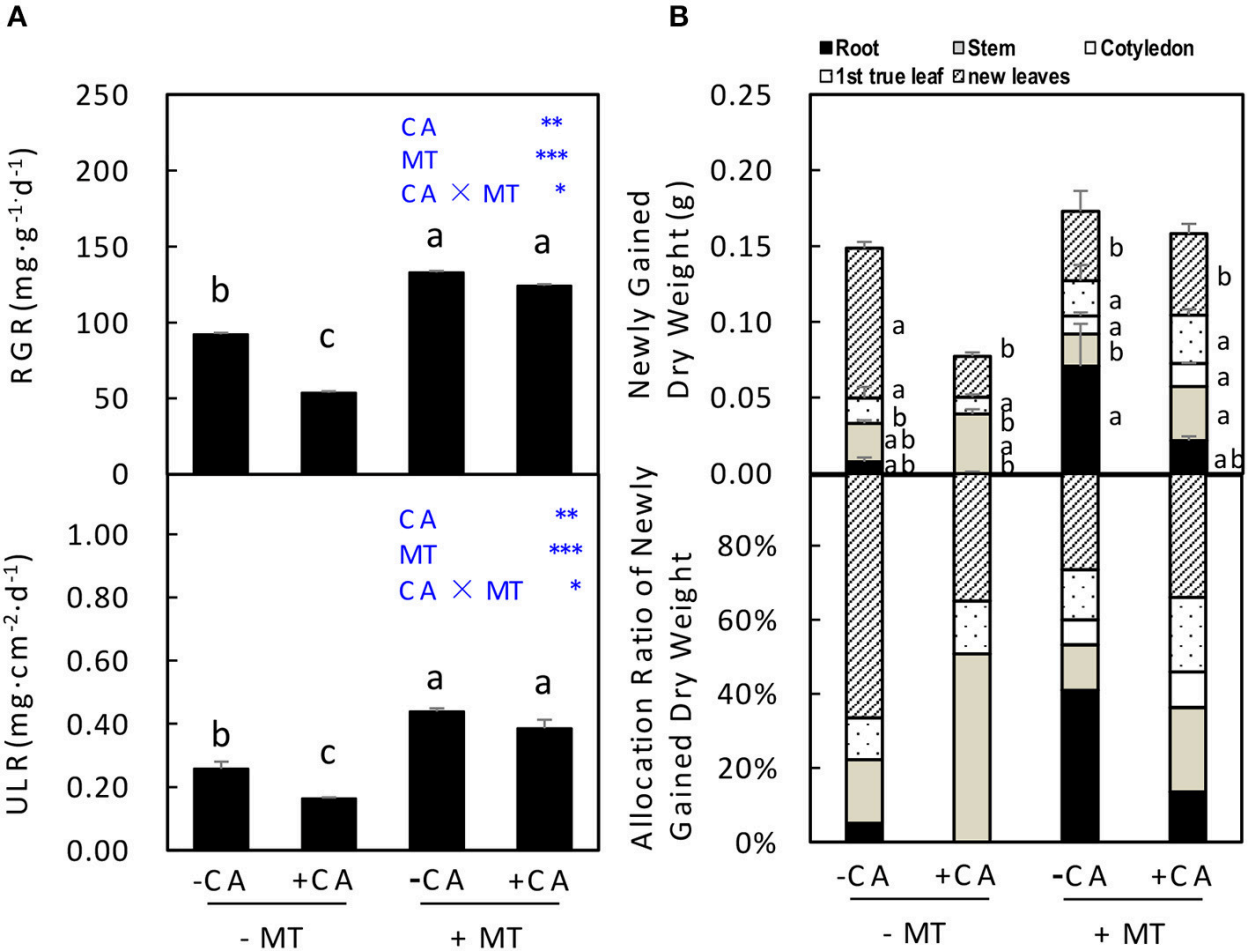
The accumulation and distribution of dry matter in cucumber seedlings as affected by MT and CA treatments. **(A)** Relative growth rate (RGR) of the total dry mass and unit leaf rate (ULR). **(B)** Newly gained dry weight and the allocation of newly gained dry weight to different organs. −MT and +MT represent application of 0 and 10 μM MT, respectively. −CA and +CA indicate application of 0 and 0.4 mM CA, respectively. Values are means ± standard error (*n* = 3). Different letters over the bars denote significance at *P* < 0.05 by Tukey's HSD-test. Light-blue letters and asterisks indicate the sources of variation: CA, cinnamic acid; MT, melatonin; CA × MT, the interaction of CA and MT. ^*^P < 0.05; ^**^P < 0.01; ^***^P < 0.001.

In experiment 2, the RGR and ULR of seedlings were significantly decreased by −MT/+CA compared to −MT/−CA (Figure 4A). However, the adverse effects of CA were efficiently reduced by MT through significantly increasing both RGR and ULR (compare +MT/+CA vs. −MT/+CA; Figure 4A). In addition, under non-MT conditions, CA significantly decreased the newly gained dry weight of seedlings through reducing dry weight allocation in new leaves (compare −MT/+CA vs. −MT/−CA), resulting in obviously lower allocation ratio of newly gained dry weight in new leaves (Figure 4B). Interestingly, however, MT significantly increased the newly gained dry weight of seedlings through increasing dry weight allocation in cotyledon but not new leaves (compare +MT/+CA vs. −MT/+CA; Figure 4B).

### Responses of plant morphology, nutrient elements, and endogenous hormone to CA stress and MT alleviation

**Figure 5:**
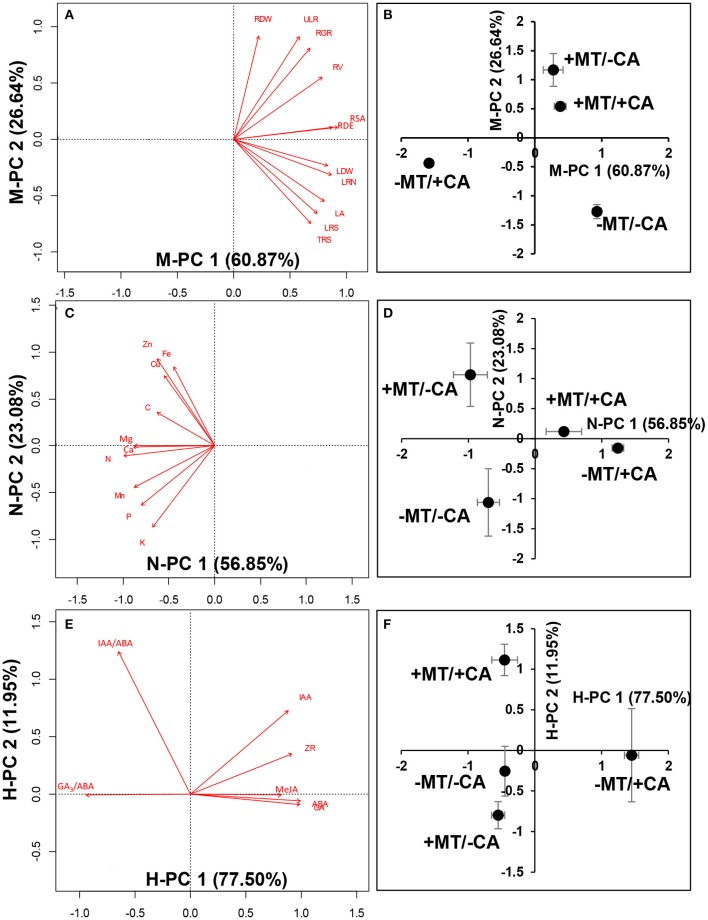
Principal component analysis (PCA) analysis of morphological characteristics **(A,B)**, nutrient elements **(C,D)**, and endogenous hormones **(E,F)**. −MT and +MT represent application of 0 and 10 μM MT, respectively. −CA and +CA indicate application of 0 and 0.4 mM CA, respectively. LA, leaf area; RDW, root dry weight; LDW, leaf dry weight; RSA, root surface area; RV, root volume; RDE, LR density/MR; LRN, 1st Order lateral root no. For definitions of these root parameters, see Table 1 and Supplementary Figure S1. Bars represent standard errors (*n* = 3). The direction and length of the red arrows indicate the correlation and its strength, respectively.

In experiment 2, the PCA analysis of plant morphology showed that the −MT/+CA treatment was clearly separated from other treatments by the first principal component (PC1, 60.87%; Figures 5A,B). The major morphological parameters contributing to PC1 indicated that MT obviously increased the root surface area and LR density/MR under CA-stress (compare +MT/+CA vs. −MT/+CA). Moreover, the treatments with MT (+MT/–CA and +MT/+CA) were clearly separated from the treatments without MT (−MT/−CA and −MT/+CA) by the second principal component (PC2, 26.64%). The major morphological parameters contributing to PC2 indicated that MT obviously increased root dry weight under CA-stress (compare +MT/+CA vs. −MT/+CA).

The PCA analysis of nutrient elements showed clear separations among the −MT/−CA, −MT/+CA, and +MT/+CA treatments along PC1 (56.85%; Figures 5C,D). The major nutrient elements contributing to PC1 indicated that MT increased the contents of N, Ca and Mg in seedlings under CA-stress (compare +MT/+CA vs. −MT/+CA).

The PCA analysis of endogenous hormones showed the −MT/+CA treatment was clearly separated from other treatments by PC1 (77.50%; Figures 5E,F). The major endogenous hormones contributing to PC1 indicated that MT obviously decreased ABA, MeJA, and GA_3_ under CA-stress (compare +MT/+CA vs. −MT/+CA). In addition, the +MT/+CA treatment was also clearly separated from the −MT/+CA treatment by PC2 (11.95%), through increasing the IAA/ABA ratio.

Moreover, strong relationships were found among most morphological parameters, nutrient elements and endogenous hormones (Table 2). For instance, the contents of N and Ca (the major parameters contributing to PC1 from PCA of nutrient elements; Figures 5C,D) were significantly positively correlated with LR density/MR (the major parameters contributing to PC1 from PCA of plant morphology; Figures 5A,B), and were significantly negatively correlated with ABA, MeJA, and GA_3_ (the major parameters contributing to PC1 from PCA of endogenous hormones; Figures 5E,F).

**Table 2:** Pearson correlation coefficients among morphological characteristics, nutrient elements, and endogenous hormones.

	**RGR**	**ULR**	**LA**	**RDW**	**LDW**	**TRS**	**RSA**	**RV**	**RDE**	**LRS**	**LRN**	**C**	**N**	**P**	**K**	**Ca**	**Mg**	**Cu**	**Fe**	**Mn**	**Zn**	**ABA**	**GA3**	**ZR**	**Me-JA**	**IAA**	**IAA/ABA**	**GA3/ABA**
RGR		0.98	n.s.	0.62	n.s.	n.s.	0.72	0.90	0.70	n.s.	n.s.	0.97	0.69	n.s.	n.s.	0.61	n.s.	n.s.	n.s.	n.s.	0.68	0.83	0.86	0.77	0.89	0.70	0.59	0.73
ULR			n.s.	0.67	n.s.	n.s.	0.62	0.84	0.65	n.s.	n.s.	0.95	0.62	n.s.	n.s.	n.s.	n.s.	n.s.	n.s.	n.s.	0.76	0.75	0.77	0.73	0.87	0.64	n.s.	0.65
LA				n.s.	0.87	0.86	0.74	n.s.	0.69	0.86	0.81	n.s.	0.73	0.76	0.92	0.64	n.s.	n.s.	n.s.	0.77	n.s.	0.70	0.68	0.63	n.s.	0.59	n.s.	0.70
RDW					n.s.	n.s.	n.s.	n.s.	n.s.	n.s.	n.s.	n.s.	n.s.	n.s.	n.s.	n.s.	n.s.	n.s.	0.95	n.s.	0.72	n.s.	n.s.	n.s.	n.s.	n.s.	n.s.	n.s.
LDW						0.65	0.75	0.58	0.81	0.64	0.78	0.61	0.73	0.62	0.87	0.65	n.s.	n.s.	n.s.	0.64	n.s.	0.77	0.77	0.77	n.s.	0.62	0.60	0.74
TRS							0.66	n.s.	n.s.	0.95	0.84	n.s.	0.63	0.81	0.84	n.s.	n.s.	n.s.	n.s.	0.74	n.s.	n.s.	n.s.	n.s.	n.s.	0.58	n.s.	0.61
RSA								0.89	0.82	0.74	0.82	0.75	0.79	0.59	0.60	n.s.	n.s.	n.s.	n.s.	0.59	n.s.	0.96	0.92	0.67	n.s.	0.79	0.70	0.94
RV									0.72	n.s.	n.s.	0.91	0.65	n.s.	n.s.	n.s.	n.s.	n.s.	n.s.	n.s.	n.s.	0.90	0.88	0.63	0.72	0.68	0.76	0.84
RDE										0.61	0.85	0.70	0.85	ns	0.62	0.75	0.60	0.65	n.s.	0.68	0.61	0.90	0.89	0.90	0.67	0.83	n.s.	0.83
LRS											0.86	n.s.	0.64	0.71	0.74	n.s.	n.s.	n.s.	n.s.	0.66	n.s.	0.64	0.58	n.s.	n.s.	0.61	n.s.	0.69
LRN												n.s.	0.79	0.68	0.73	0.67	n.s.	n.s.	n.s.	0.72	n.s.	0.79	0.76	0.69	n.s.	0.79	n.s.	0.78
C													0.68	n.s.	n.s.	n.s.	n.s.	n.s.	n.s.	n.s.	0.60	0.83	0.84	0.71	0.84	0.69	0.59	0.75
N														0.83	0.71	0.86	0.77	n.s.	n.s.	0.87	n.s.	0.87	0.87	0.85	0.60	0.94	n.s.	0.82
P															0.86	0.66	0.64	n.s.	n.s.	0.92	n.s.	n.s.	n.s.	n.s.	n.s.	0.70	n.s.	n.s.
K																0.59	n.s.	n.s.	n.s.	0.82	n.s.	n.s.	n.s.	0.58	n.s.	n.s.	n.s.	n.s.
Ca																	0.77	n.s.	n.s.	0.71	n.s.	0.69	0.77	0.86	0.73	0.80	n.s.	0.58
Mg																		n.s.	n.s.	0.74	0.59	n.s.	n.s.	0.76	0.59	0.62	n.s.	n.s.
Cu																			n.s.	n.s.	0.81	n.s.	n.s.	0.58	n.s.	0.61	n.s.	n.s.
Fe																				n.s.	0.68	n.s.	n.s.	n.s.	n.s.	n.s.	n.s.	n.s.
Mn																					n.s.	0.62	0.63	0.65	n.s.	0.70	n.s.	0.59
Zn																						n.s.	n.s.	0.67	0.66	0.58	n.s.	n.s.
ABA																							0.97	0.83	0.70	0.87	0.66	0.96
GA3																								0.85	0.76	0.84	0.66	0.88
ZR																									0.84	0.82	n.s.	0.75
MeJA																										0.61	n.s.	0.58
IAA																											n.s.	0.87
IAA/ABA																												0.60
GA3/ABA																												

*Data was collected from seedlings grown in different treatments. Blue color indicated a negative correlation at the P < 0.05 level between parameters. n.s. Not significant. LA, leaf area; RDW, root dry weight; LDW, leaf dry weight; RSA, root surface area; RV, root volume; RDE, LR density/MR; LRN, 1st Order LR no. (Further definitions are included in Table 1)*.

## Discussion

Exogenous application of melatonin has become an increasingly popular means to active plants defense mechanisms under stressful environmental conditions. However, little information is available concerning the role of MT in alleviating CA stress in plants. The present study provides evidence that the exogenous application of MT is a potential solution for improving cucumber seedling growth under CA-stress by regulating the morphological growth, and the contents of nutrient elements and endogenous hormones. Although CA inhibited cucumber seedling growth by negatively influencing most plant parameters, such as, leaf area (Figure 2), root length (Figure 3), and dry weight (Figure 4), these inhibitory effects of CA were effectively alleviated by MT application.

In this study, several lines of evidence suggested that exogenous application of MT could improve cucumber seedling growth under CA stress. First, MT application improved morphological characteristics of cucumber seedlings exposed to CA stress. These improvements were mainly achieved by MT through protecting leaves from wilting (Figure 1), stimulating leaf expansion (Figure 2), and enhancing root growth (Figure 3). Similarly, a higher health index in MT-treated tomato seedlings and a larger leaf area in MT-treated maize seedlings subjected to drought stress were observed by Liu J. L. et al. (2015) and Ye et al. (2016). According to these authors, a higher health index and leaf area enabled plants to maintain high photosynthetic capacity. Generally, roots directly reflect susceptibility or tolerance to abiotic stresses in plants. A well-developed root system can withstand adverse effects of environmental stresses (Wang et al., 2006; Arnao and Hernández-Ruiz, 2015). In the present study, MT application increased the number and diameter of lateral roots under CA stress (Figure 3). This finding is consistent with previous results (Zhang et al., 2013) in which lateral root number in cucumber seedlings was increased by MT application under water stress. Since most root morphology-related parameters (Table 1) were obviously decreased by CA under non-MT conditions but were not affected under MT conditions (Figure 3A), the improvements in root growth and development might be a major factor attributing to the alleviation of CA-stress by MT. Indeed, root morphology is an important developmental and agronomic trait that strongly influences nutrient uptake, abiotic stress resistance, and overall plant growth and development (Jung and McCouch, 2013). An overall improvement due to MT application was observed not only in our cucumber seedlings subjected to CA-stress (Figures 1–3), but also in maize seedlings subjected to salt stress (Jiang et al., 2016), tomato seedlings subjected to drought stress (Liu J. L. et al., 2015), and cucumber seedlings subjected to water stress (Zhang et al., 2013).

Second, MT application enhanced nutrient uptake by plants, especially for N, Ca, and Mg (Figures 5C,D). Among them, N is an essential mineral element that is required in the greatest amount in plants (Maathuis, 2013). The foremost function of N is to provide amino groups in amino acids, the building blocks of every protein (Maathuis, 2013). In the present work, the total N content in cucumber seedlings was increased by MT application under CA stress (Figures 5C,D). Since the total N content was strongly positively correlated with most plant morphological parameters, such as, relative growth rate, unit leaf rate, leaf dry weight, and root surface area (Table 2), N was probably one of the major nutrient elements responsible for plant morphological changes induced by MT under CA treatments. In addition to N, the Ca and Mg contents in cucumber seedlings were also increased by MT application under CA stress (Figures 5C,D). Similar results were observed in MT-pretreated cucumber seedlings under nitrate stress (Zhang et al., 2017). Ca is generally thought to have a crucial function in stabilizing cell walls and membranes (Xu et al., 2014) and is recognized as a signal in abiotic stress (Kopittke, 2012), while Mg plays an important role in plant growth and development, such as, chlorophyll biosynthesis (Hansson et al., 2013). Enhanced Ca and Mg contents in MT-treated seedlings suggested that MT might have achieved its protective effects on plant growth and development (Turk and Erdal, 2015). This could be partly supported by the positive relationships between the Ca and Mg contents and LR density/MR (Table 2), a major root morphological parameter improved by MT under CA-stress (Figures 5A,B). Moreover, MT application significantly increased the C content in the whole seedling under CA stress (Figures 5C,D), suggesting that MT might enhance aboveground C assimilation under CA-stress. Indeed, MT significantly increased the dry matter accumulation of seedlings under CA stress (Figure 4).

Third, MT improved seedling growth under CA stress by regulating endogenous hormone levels. Obviously, MT decreased the contents of ABA, MeJA, and GA_3_ in seedlings under CA-stress (Figures 5E,F). More importantly, these endogenous hormones were strongly negatively correlated with most plant morphological parameters, such as, RGR, ULR, root volume, and LR density/MR (Table 2). Among these endogenous hormones, ABA is often utilized as a stress signal, which often accumulates under stressful conditions (Fujita et al., 2011). Since MT reduced the ABA content in the 2nd true leaf under CA stress (Supplementary Figure S2), MT might efficiently alleviate CA stress in seedlings. In addition to the absolute amount, the balance between endogenous hormones might play an important role in the process by which MT alleviated CA stress (Peleg and Blumwald, 2011; Ha et al., 2012). We noted that the IAA/ABA and GA_3_/ABA ratios, which were strongly positively related to most plant morphological parameters (e.g., RGR, leaf dry weight, root surface area, and root volume; Table 2), were obviously increased by MT under CA stress (Figures 5E,F). Similar results were obtained in plants under other abiotic stresses such as, salinity and cold stress (Huang et al., 2015; Pompeiano et al., 2016).

Although the exogenous application of MT had a potential to improve cucumber seedling growth under CA-stress, the effects of MT on seedling growth were strongly concentration-dependent. Chen et al. (2009) demonstrated that MT stimulated *Brassica juncea* growth at 0.1 μM but inhibited growth at 100 μM. In this study, under non-CA stress (0 mM CA), cucumber seedling growth were suppressed by 10 μM MT (Figure 1). However, when seedling pretreated with 10 μM MT were subsequently subjected to CA stress (0.4 mM CA), the negative effects of CA on seedling growth were efficiently alleviated (Figure 1). This result suggests that the combination of two unfavorable conditions may exert a beneficial effect on plant growth.

In summary, cucumber seedling growth was generally inhibited under CA stress. Melatonin rescued cucumber seedling growth under CA stress by improving morphological characteristics, enhancing nutrient uptake, and regulating endogenous hormone levels. However, the findings presented here represent only the beginning of research on the use of exogenous MT to restore cucumber seedling growth under CA stress, and further research on the underlying physiological and molecular mechanisms is needed.

## Author contributions

Conceived and designed the experiments: JL, YT, MQ, and LG. Performed the experiments: JL and YL. Analyzed the data: JL and YT. Wrote the paper: JL, YL, YT, and WZ.

### Conflict of interest statement

The authors declare that the research was conducted in the absence of any commercial or financial relationships that could be construed as a potential conflict of interest.

## Supplementary material

Supplementary Figure1 Schematic of root morphological characteristics. Color-coded schematic main root (MR), 1st Order lateral roots (1st Order LRs), and 2nd Order LRs are shown in red, blue, and yellow, respectively.

Supplementary Figure2 Endogenous hormone contents in different plant organs as affected by MT and CA treatments. Endogenous hormones, including ABA, IAA, GA_3_, ZR, and MeJA, in the cucumber root, stem, cotyledon, 1st true leaf, and 2nd true leaf. 0 d, before CA supplementation; 5 d, five days after CA supplementation in liquid solution; −MT and +MT represent application of 0 and 10 μM MT, respectively. −CA and +CA indicate application of 0 and 0.4 mM CA, respectively. Values are means ± standard error (*n* = 3). Different letters over the bars denote significance at *P* < 0.05 by Tukey's HSD-test.
