# Supplementary figures and images for: Melatonin Has the Potential to Alleviate Cinnamic Acid Stress in Cucumber Seedlings
Source: Front Plant Sci. 2017 Jul 13;8:1193. doi: 10.3389/fpls.2017.01193 (PMC5508022; doi:10.3389/fpls.2017.01193)

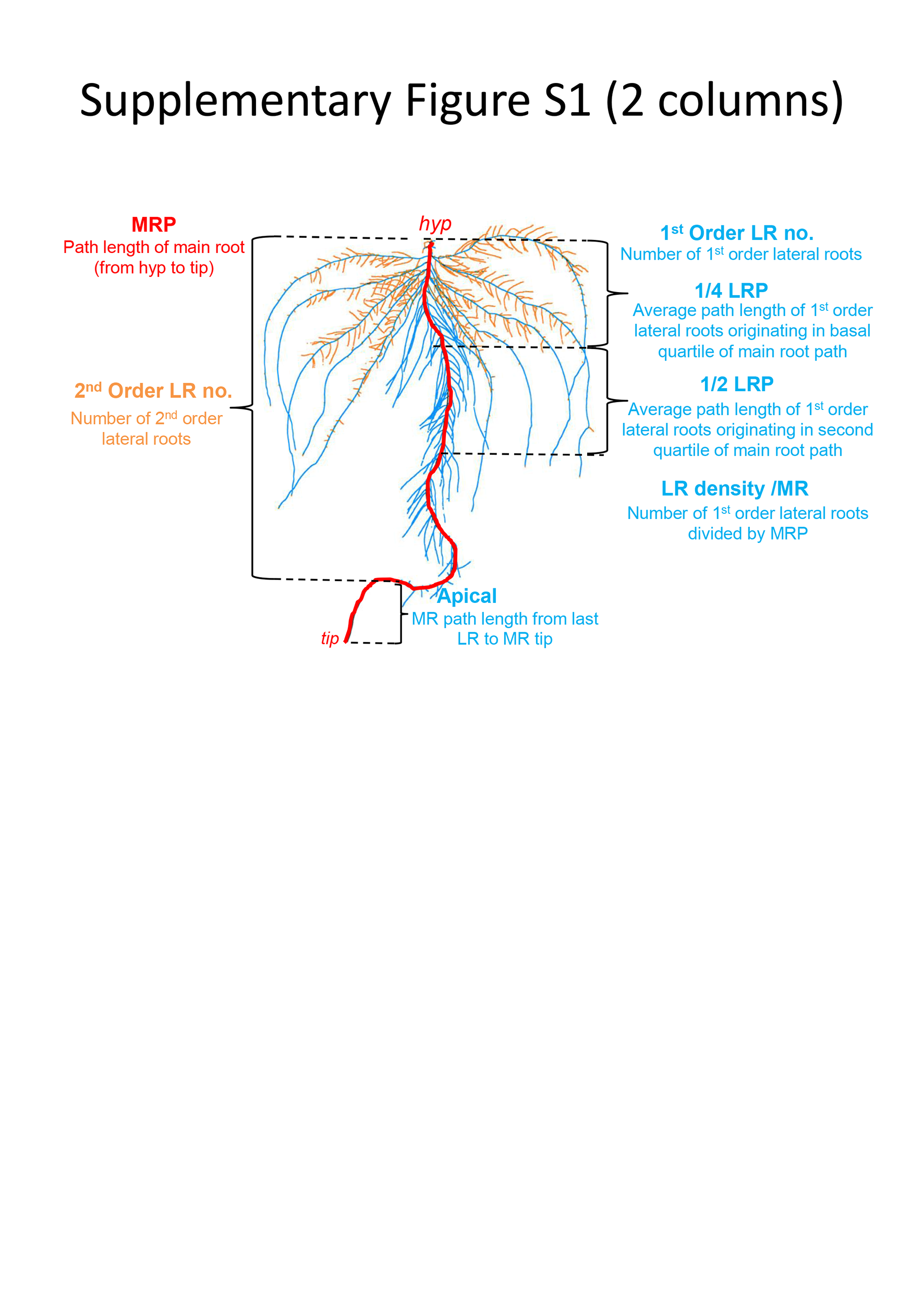

Supplement: Supplementary Figure1 — Schematic of root morphological characteristics. Color-coded schematic main root (MR), 1st Order lateral roots (1st Order LRs), and 2nd Order LRs are shown in red, blue, and yellow, respectively. [file Image1.TIF]

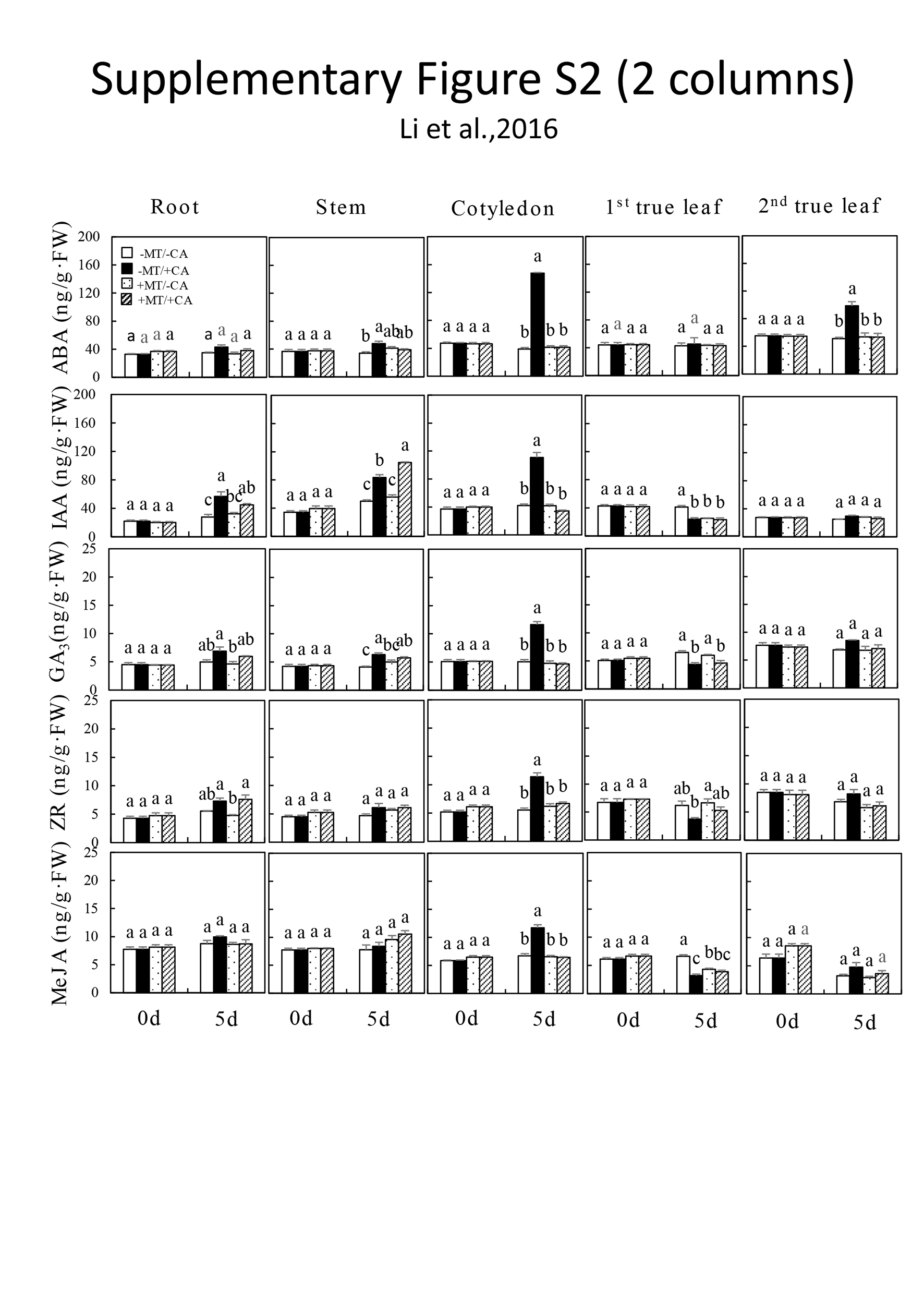

Supplement: Supplementary Figure2 — Endogenous hormone contents in different plant organs as affected by MT and CA treatments. Endogenous hormones, including ABA, IAA, GA3, ZR, and MeJA, in the cucumber root, stem, cotyledon, 1st true leaf, and 2nd true leaf. 0 d, before CA supplementation; 5 d, five days after CA supplementation in liquid solution; −MT and +MT represent application of 0 and 10 μM MT, respectively. −CA and +CA indicate application of 0 and 0.4 mM CA, respectively. Values are means ± standard error (n = 3). Different letters over the bars denote significance at P < 0.05 by Tukey's HSD-test. [file Image2.TIF]
